# Molecular Mechanisms of Mast Cell Activation by Cholesterol-Dependent Cytolysins

**DOI:** 10.3389/fimmu.2021.670205

**Published:** 2021-06-23

**Authors:** Lubica Draberova, Magda Tumova, Petr Draber

**Affiliations:** Department of Signal Transduction, Institute of Molecular Genetics of the Czech Academy of Sciences, Prague, Czechia

**Keywords:** mast cell, cholesterol-dependent cytolysins, pore-forming toxins, Ca^2+^ signaling, cytokine production, streptolysin O, pneumolysin, listeriolysin O

## Abstract

Mast cells are potent immune sensors of the tissue microenvironment. Within seconds of activation, they release various preformed biologically active products and initiate the process of *de novo* synthesis of cytokines, chemokines, and other inflammatory mediators. This process is regulated at multiple levels. Besides the extensively studied IgE and IgG receptors, toll-like receptors, MRGPR, and other protein receptor signaling pathways, there is a critical activation pathway based on cholesterol-dependent, pore-forming cytolytic exotoxins produced by Gram-positive bacterial pathogens. This pathway is initiated by binding the exotoxins to the cholesterol-rich membrane, followed by their dimerization, multimerization, pre-pore formation, and pore formation. At low sublytic concentrations, the exotoxins induce mast cell activation, including degranulation, intracellular calcium concentration changes, and transcriptional activation, resulting in production of cytokines and other inflammatory mediators. Higher toxin concentrations lead to cell death. Similar activation events are observed when mast cells are exposed to sublytic concentrations of saponins or some other compounds interfering with the membrane integrity. We review the molecular mechanisms of mast cell activation by pore-forming bacterial exotoxins, and other compounds inducing cholesterol-dependent plasma membrane perturbations. We discuss the importance of these signaling pathways in innate and acquired immunity.

## Introduction

Mast cells are critical initial players in innate and acquired immunity and, in this way, ultimately influence the outcome of various diseases. They are dispersed throughout the body tissues, but most abundantly are found at host-environment interfaces such as the skin, respiratory tract, and oral/gastrointestinal mucosa, suggesting their role as sentinels of infection. Mast cells share some features with other immune effector cells, such as basophils, macrophages, and neutrophils. However, they differ by their unique interactions with blood vessels and capacity to rapidly, within seconds and minutes, release an extensive set of inflammatory mediators such as histamine, proteases, lipid mediators, cytokines, and chemokines. Like other cell types of the immune system, mast cells communicate with their environment predominantly *via* surface receptors recognizing various soluble or membrane-bound ligands. Depending on mast cell location and the overall context, mast cell activation leads to activation of multiple immune effector mechanisms, cell differentiation, chemotaxis, or inhibition of ongoing immune reactions. Various mast cell immune functions have been summarized in several reviews (1–5).

Mast cells can be activated by engagement of various plasma membrane receptors. The most studied receptor on the surface of mast cells is the high-affinity IgE receptor (FcϵRI) associated with the mast cells’ role in pathological conditions such as allergy, anaphylaxis, and asthma. Besides FcϵRI, many other surface receptors recognize a variety of soluble or membrane-bound ligands that tightly upregulate or downregulate mast cell responsiveness. Extensively studied are pattern recognition receptors such as Toll-like receptors (TLRs), which are activated in response to conserved pathogen-associated molecular patterns (6, 7), and the Mas-related G protein-coupled receptor (Mrgpr) family, especially Mrgprb2 in the mouse and its human ortholog MRGPRX2 (8–10). Many other receptors present on the mast cell surface have been covered in numerous reviews (2, 4, 5, 11). Some of the plasma membrane receptors allow the innate system to identify invading bacteria and even viruses by their expression of pathogen-associated molecular patterns and allow mast cells to directly respond to bacteria by degranulation and production of *de novo* synthesized proinflammatory and anti-inflammatory products (3, 12–17). Thus, there is an intricate network of surface receptors and regulatory proteins that can either induce mast cell activation or inhibit mast cell signaling.

There is a widespread notion that mast cells are part of the antimicrobial host defense. This is based on experiments showing that mast cell-deficient mice are more sensitive to bacterial infection than WT mice (13, 18–20). In experiments with *Listeria monocytogenes*, mast cell-deficient mice showed approximately hundred-fold higher bacterial burden and significantly impaired neutrophil mobilization when compared to control mice. Although bacteria bound to mast cells triggered degranulation, their phagocytosis was negligible. Thus, mast cells control bacterial infection not through bacterial uptake, but by activation and rapid degranulation associated with the release of pre-synthesized pro-inflammatory mediators, cytokines and chemokines, which cause influx of other immune cells, mainly neutrophils, to the site of infection. Once recruited, neutrophils not only phagocytose and destroy bacteria, but also become activated and secrete inflammatory mediators, hence amplifying the anti-bacterial inflammatory response (21). Other studies showed that animals lacking mast cells or mast cell signaling molecules respond differentially to bacterial infection when compared with wild type controls. Interestingly, in response to bacteria, mast cells, in contrast to other cell types such as macrophages, elicit only a proinflammatory response but not the type I interferon (IFN-I) response. It has been found that this phenomenon could be attributed to the spatial regulation of proinflammatory and IFN-I responses from different subcellular sites; proinflammatory responses occur from the cell surface, whereas IFN-I responses are induced from endolysosomal compartments (22). This review focuses on the molecular mechanisms of mast cell activation by cholesterol-dependent cytolysins (CDCs).

## CDCs

CDCs are a class of pore-forming proteins produced by a wide range of predominantly Gram-positive bacteria. They form the most prominent toxin family, comprising at least 28 bacterial species, that mediate bacterial virulence. The most frequently studied CDCs are those produced by pathogenic *Streptococci*, *Listeria*, and *Clostridia*. The summary of cytolysins discussed in this review, their bacterial producers, and a subset of diseases they cause are presented in **Table 1**. Various strategies have been developed to eliminate the pathogens producing CDCs. One effective way is sensing CDCs produced by the pathogens by mast cells followed by their activation and mobilizing innate and adaptive immunity mechanisms. In terms of their effects on mast cells, extensively studied CDCs were those produced by pathogenic *Streptococci* and *Listeria* (see below). Numerous studies have shown that the CDCs’ pore-forming ability requires the presence of cholesterol in the plasma membrane of host cells (38–47).

**Table 1:** Summary of cytolysins discussed in this review, their bacterial producers, and a subset of associated diseases.

Toxin	Abbreviation	Bacteria	Diseases
Streptolysin O	SLO	*Streptococcus pyogenes*	Various infectious diseases as pharyngitides, rheumatic fever, scarlet fever, necrotizing soft tissue infection, toxic shock syndrome (23, 24)
Pneumolysin	PLY	*Streptococcus pneumoniae*	Bacterial pneumonia, otitis media, bacterial meningitis (25, 26)
Listeriolysin O	LLO	*Listeria monocytogenes*	Listeriosis (manifestations include abortion, sepsis, meningoencephalitis, febrile gastroenteritis syndrome) (27, 28)
Streptolysin S^*^	SLS	*Streptococcus equi*	Disease of the upper respiratory tract and associated lymph nodes in equids (29, 30)
Perfringolysin	PFO	*Clostridium perfringens*	Histotoxic infections, pathogenesis of gas gangrene (31, 32)
Vaginolysin	VLY	*Gardnerella vaginalis*	Bacterial vaginosis (33)
Lectinolysin	LLY	*Streptococcus mitis*	Infective endocarditis, bacteremia and septicemia (34)
		*Streptococcus pseudopneumoniae*	
Suilysin	SLY	*Streptococcus suis*	Meningitis (35)
Intermedilysin	ILY	*Streptococcus intermedius*	Associated with brain and liver abscesses (36)

*SLS was also identified in S. pyogenes and most of other group A Streptococcus isolates (37). However, the hemolytic activity of SLS is not affected by cholesterol (29).

### General Mechanism of Plasma Membrane Pore Formation by CDCs

**Figure 1:**
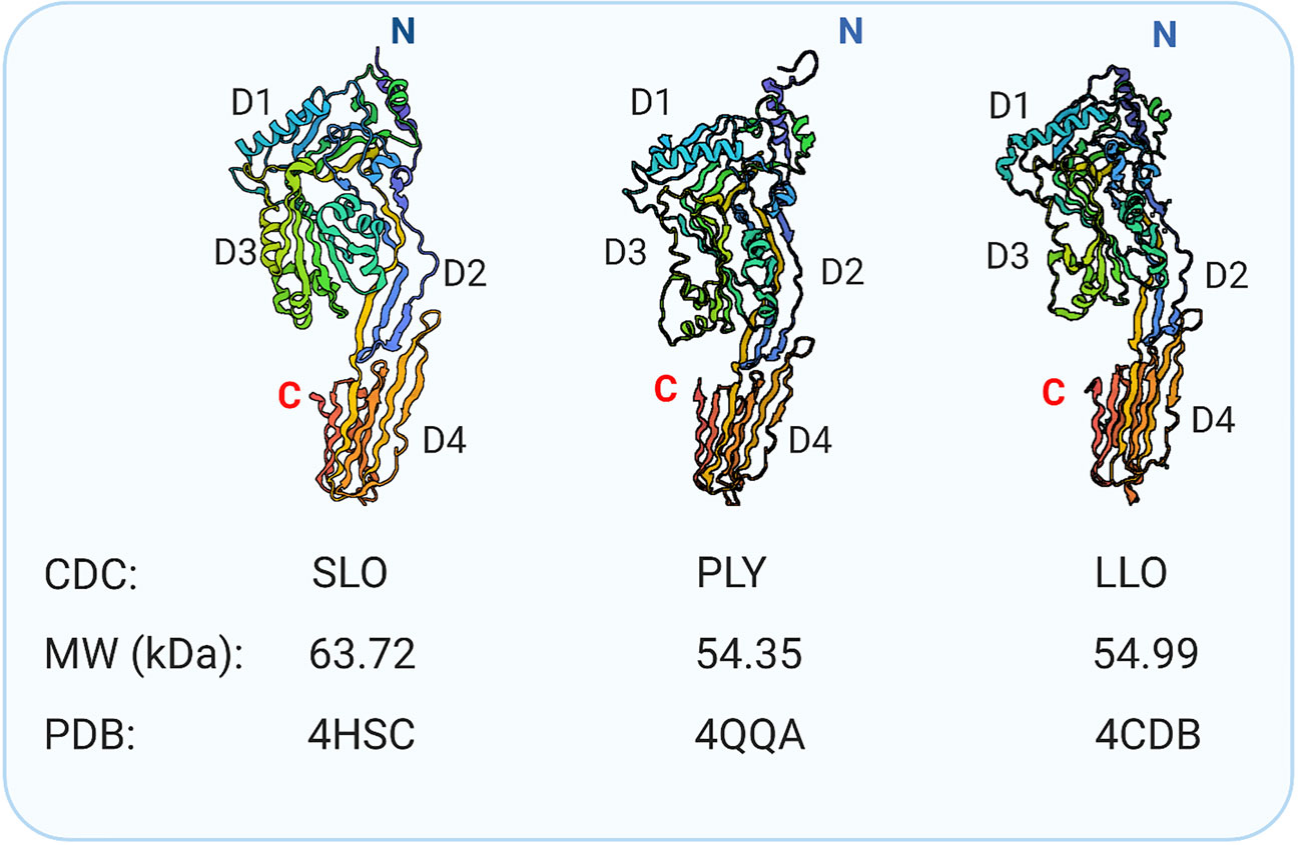
Bacterial pore-forming cholesterol-dependent cytolysins used in mast cell research. Crystal structure of SLO (42), PLY (49), and listeriolysin (LLO) (50). Indicated are N-terminus, C-terminus, and four domains rich in β-sheets: Domain 1 (D1), Domain 2 (D2), Domain 3 (D3) with the transmembrane spanning region, and Domain 4 (D4) involved in the initial direct interaction with cholesterol and glycans (51–53). The molecular weights and protein data bank (PDB) codes are also indicated.

**Figure 2:**
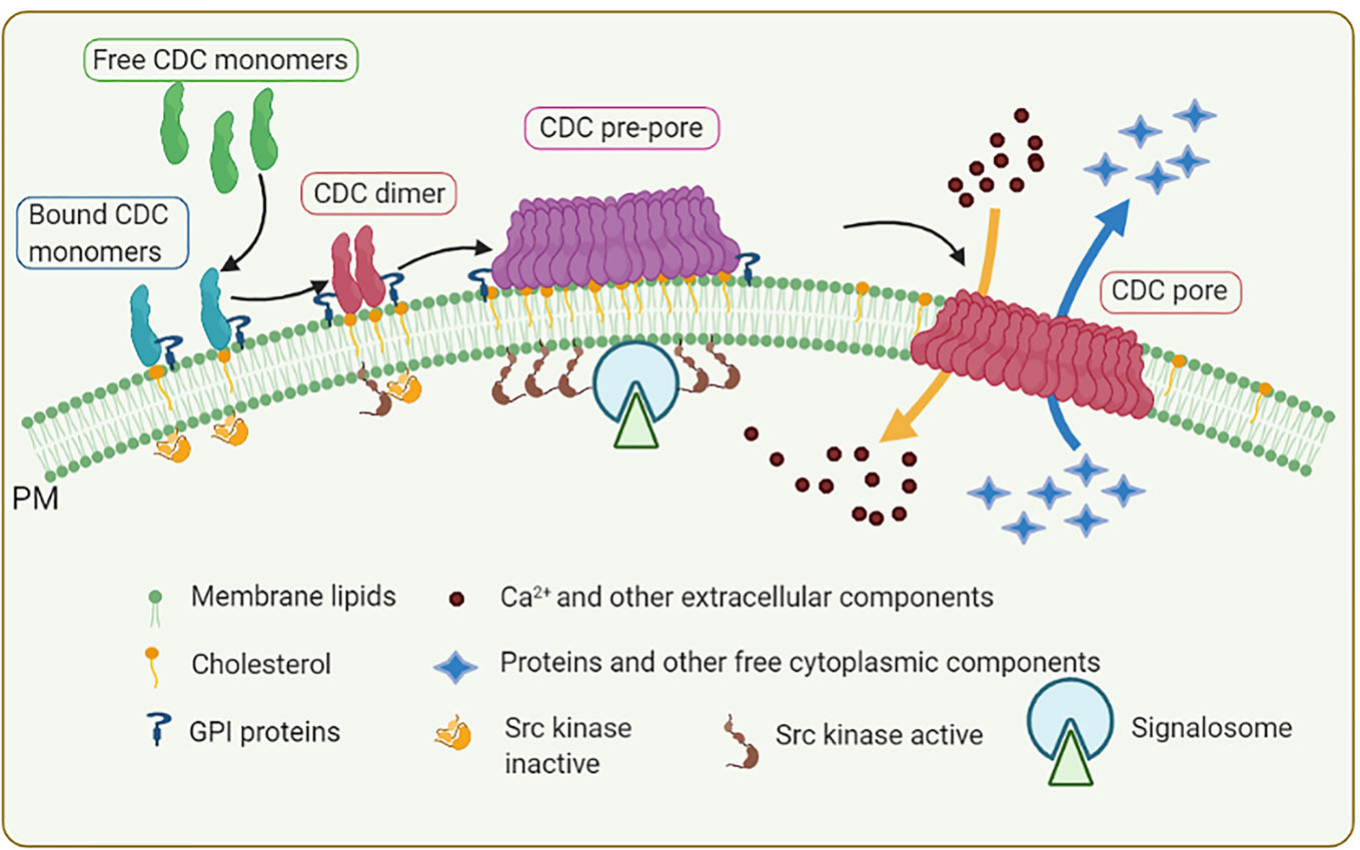
Formation of membrane pores by CDCs. CDC monomers released from bacteria bind through their D4 domain to plasma membrane (PM) microdomains called lipid rafts enriched in cholesterol, GPI-anchored proteins, and Src family kinases. PM-bound monomers form dimers that polymerize into pre-pore structures containing 30 to 50 CDC monomers. In this process, CDCs induce aggregation of lipid raft components leading to formation of signaling assemblies called signalosome. The signalosomes are capable to initiate cell activation events. Individual CDCs in the pre-pore structure undergo conformational rearrangement and formation of membrane-spanning β-strands, leading to concerted membrane insertion and formation of the pore with approximately 25 nm in diameter. Formation of the transmembrane pore results in influx of Ca^2+^ into the cytoplasm and efflux of K^+^, other small molecules (e.g., ATP), and proteins through the plasma membrane. These processes trigger various cell responses involved in repairing the plasma membrane and activating innate and acquired immunity.

Formation of the plasma membrane pores by CDCs relies on the self-assembly of monomers bound individually to cholesterol-rich membranes, followed by dimerization and oligomerization. At the protein level, CDCs are conserved across multiple organisms (46, 48). Structurally, various CDCs are organized into four functional domains. **Figure 1** shows crystal structures of three CDCs [streptolysin O (SLO) from *Streptococcus pyogenes*, pneumolysin (PLY) from *S. pneumoniae*, and listeriolysin O (LLO) from *Listeria monocytogenes*] used in mast cell research. Domains 1 and 2 retain the contact with the aqueous environment during the pore formation. Domain 3 is composed of two transmembrane helices that convert to β-strands involved in penetrating the host membrane. Domain 4 is involved in cholesterol sensing and membrane binding. This domain, which shows the highest conservation across CDCs, consists of a β-sandwich linked by structural loops and a tryptophan-rich undecapeptide (TR-UDP). A previous study has shown that the threonine-leucine pair of sequential amino acids binds to the hydroxyl group of cholesterol in cholesterol-rich membrane regions (54), but not free cholesterol (55). Thus, the threonine-leucine pair of CDC recognizes specific features of cholesterol at the plasma membrane to initiate the cholesterol-dependent interaction of the CDC with the cell. Although different CDCs have an identical cholesterol-binding motif, they exhibit different binding parameters depending on the lipid (56) and glycan (57, 58) environment. These interactions anchor the CDC monomer in a perpendicular orientation to the membrane surface. The domain 4-lipid interaction triggers conformational changes in spatially distant domain 3, which exposes a previously hidden interface involved in oligomerization and, hence, formation a pre-pore complex. Then, two sets of short α-helices in domain 3 undergo an α-helix to β-sheet transition, leading to creation of two pore-forming transmembrane β-hairpins (TMHs) per monomer, which are still above the membrane surface. Through this process, plasma membrane-bound monomers oligomerize into pre-pores consisting of 35 - 50 monomers sitting on the cell surface (**Figure 2**). Next, the pre-pore components undergo further restructuring, including shape changes, bringing domain 3 and its TMH regions to the membrane proximity. This leads to refolding the transmembrane helices into β-strands and forming a β-barrel pore in the plasma membrane with a diameter of about 30 nm (43, 45, 46, 48, 59–62).

Recent data (57, 58) showed that of eight CDCs studied [SLO, PLY, LLO, perfringolysin (PFO) from *Clostridium perfringens*; vaginolysin (VLY) from *Gardnerella vaginalis*; lectinolysin (LLY) from *Streptococcus mitis* or *S. pseudopneumoniae*; suilysin (SLY) from *S. suis*, and intermedilysin (ILY) from *S. intermedius*], all had high-affinity lectin activity that identified glycans as candidate cellular receptors. Some of the CDCs, including SLO, VLY, and PFO, bound multiple glycans, while PLY, LLY, LLO recognized a single glycan class. All of the glycans functioning as CDC receptors are found as glycolipids, transmembrane glycoproteins, or GPI-qanchored (CD-59) glycoproteins that are frequently associated with the periphery of cholesterol-enriched lipid rafts (63). Further investigation showed no competition between cholesterol and glycan receptor binding, indicating that cholesterol and glycans bind to CDCs independently. Significantly, addition of an exogenous carbohydrate receptor for each CDC inhibited the toxin activity. Thus, binding to both the glycan receptor and cholesterol-rich membrane seems to be essential to the toxic effect of the CDCs. The combined data indicated that glycoprotein and/or glycolipid receptors present on the cellular membrane contribute to the CDCs’ cell and tissue tropism.

### Cell Response to CDC in General

**Figure 3:**
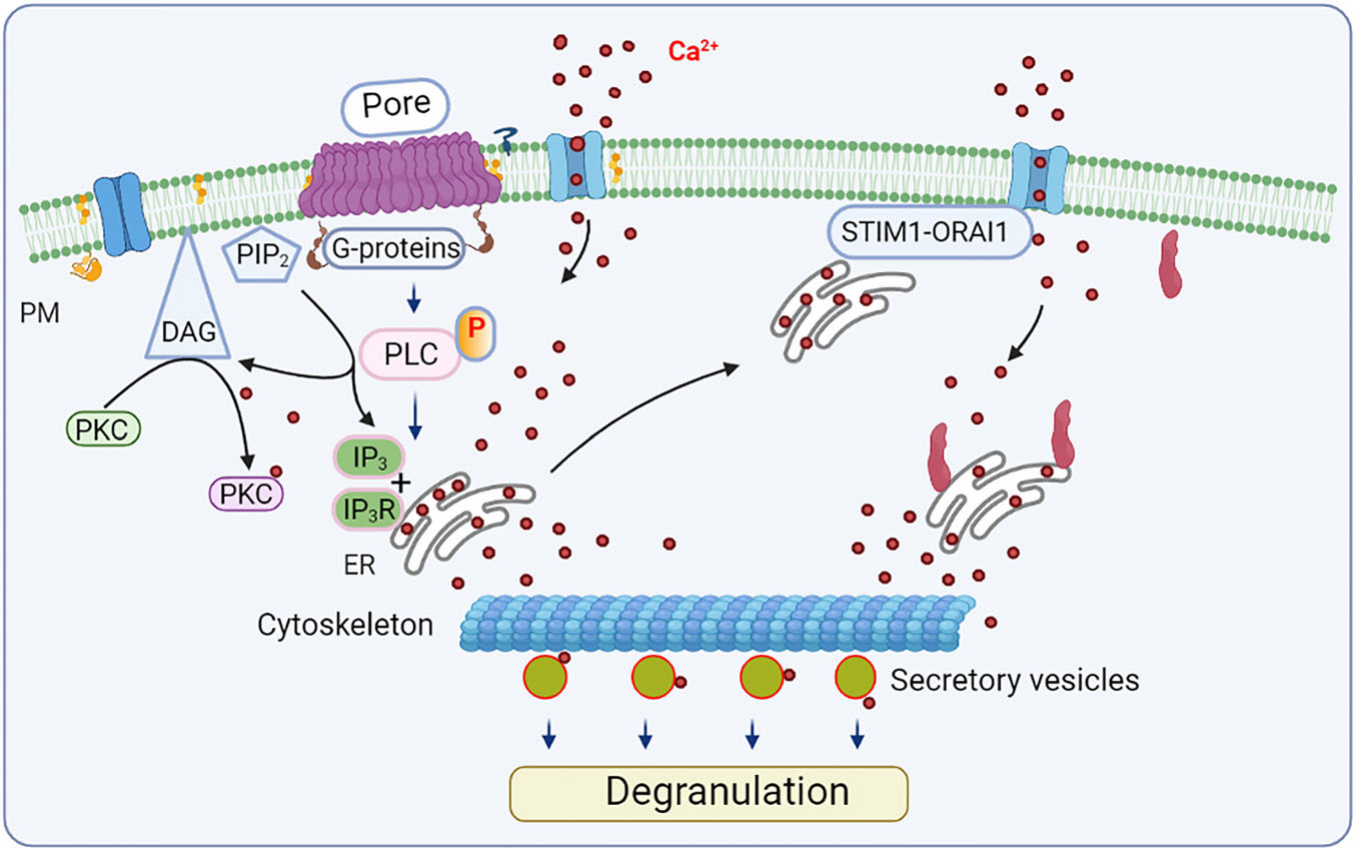
Mast cell activation by CDCs - calcium response and degranulation. Exposure of mast cells to sublytic concentrations of CDCs leads to aggregation of lipid rafts and transmembrane insertion of CDC complexes, resulting in phosphorylation of signal transduction molecules, including phospholipase C (PLC). PLC hydrolyses PM-localized phosphatidylinositol 4,5-bisphosphate (PIP2), producing inositol-1,4,5-trisphosphate (IP3) and diacylglycerol (DAG). IP3 binds to IP3 receptor (IP3R) on the endoplasmic reticulum (ER), where it stimulates the release of calcium into the cytoplasm. Free cytoplasmic calcium together with DAG activates protein kinase C (PKC). The reduced Ca^2+^ level in ER is sensed by stromal interaction molecule 1 (STIM 1), which then binds to and activates the store-operated ORAI1 calcium ion channel in the PM. Calcium could also be released due to the injury of intracellular Ca^2+^ stores by CDCs. Increased levels of free cytoplasmic Ca^2+^ and other activation events lead to the release of secretory vesicles (degranulation) in which the cytoskeleton plays an important role.

**Figure 4:**
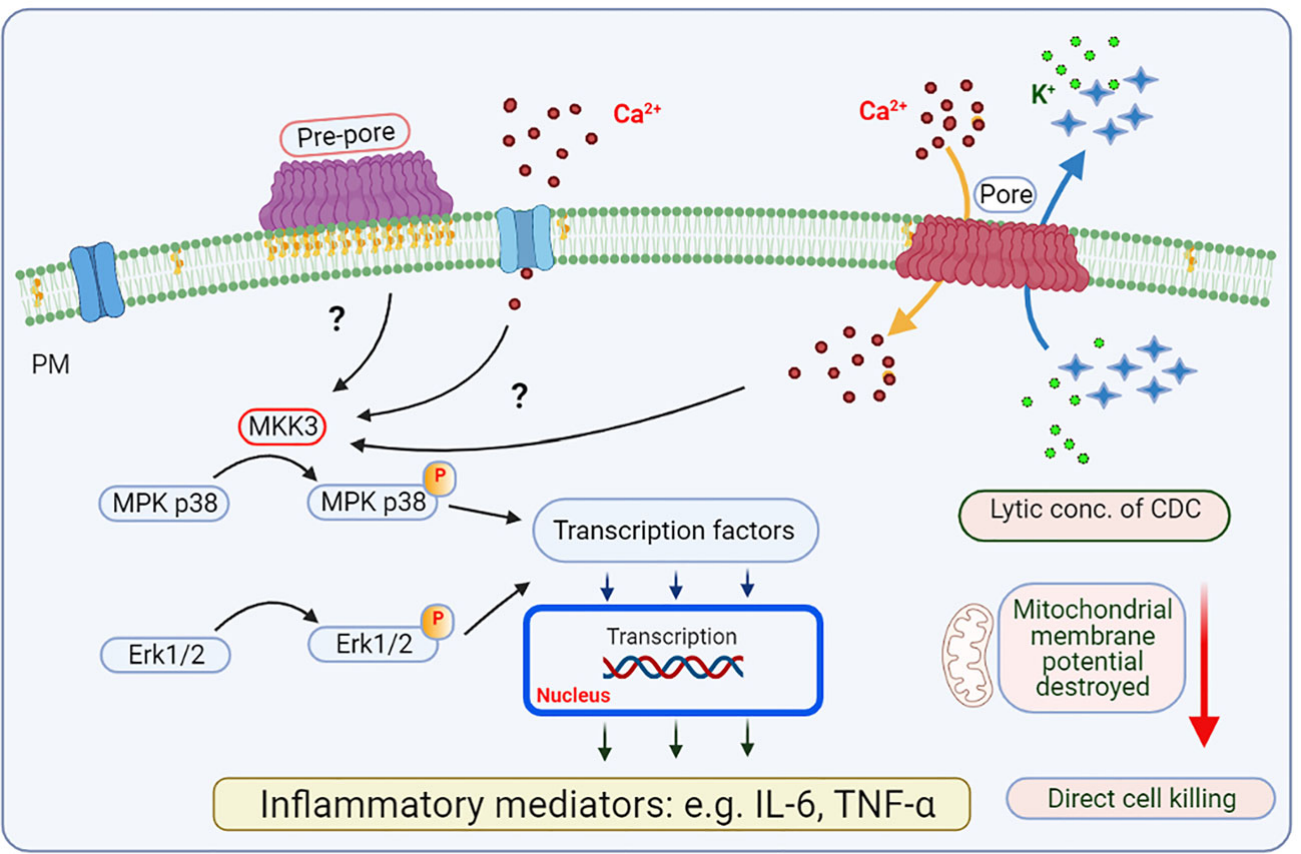
Mast cell activation by CDCs - de novo production of inflammatory mediators. Among proteins phosphorylated and activated by CDC-induced changes is mitogen-activated protein kinase (MPK)3, which is involved in phosphorylation and activation of MPK p38 and Erk1/2. These enzymes are involved in activation of transcription factors regulating transcription of selected genes for inflammatory cytokines and chemokines (e.g., IL-6 and TNF-α). It is not clear whether CDCs at the pre-pore stage have any role in these signaling events. Higher concentrations of CDCs lead to killing of target cells in the absence of production of inflammatory mediators.

The cell response to CDC depends on the cell type examined, type of CDC, and its concentration. Sensitivity to the toxic effect of CDC reflects, in part, the cell ability to repair membrane disruptions. This probably explains why erythrocytes are more sensitive to SLO than nucleated mammalian cell lines (64, 65). When used at sublytic concentrations, as is often the case *in vivo*, CDCs trigger several cellular processes, including membrane repair and resealing of the membrane pores by various mechanisms. The cell ability to reseal a limited number of pores generated by CDCs is generally dependent on Ca^2+^ levels (66). Changes in the concentration of free cytoplasmic Ca^2+^ could lead to activation of proinflammatory transcriptional regulators, including nuclear factor (NF)-κB, c-Jun N-terminal kinase (JNK), and NFAT (**Figures 3** and **4**). Various transcriptional regulators could be activated depending on the calcium signal amplitude and duration (67). Initial studies suggested that the CDCs’ Ca^2+^ signaling is mainly due to Ca^2+^ influx from the extracellular milieu (68, 69). However, later studies showed that CDCs could also induce Ca^2+^ release from intracellular stores *via* at least two independent mechanisms. The first one induces activation of intracellular Ca^2+^ channels and involves phospholipase (PLC)-inositol triphosphate receptor (IP3R)-operated Ca^2+^ channels activated *via* G-proteins and protein tyrosine kinases. The second one is Ca^2+^ channel independent, involving injury of intracellular Ca^2+^ stores such as endoplasmic reticulum (ER) (70).

Membrane repair after exposure to sublytic concentrations of CDCs is usually executed through microvesicular shedding and endocytosis; this could lead to removing the toxin from the membrane (71). Using SLO mutants with engineered defects in pore formation or oligomerization, the authors found that oligomerization, in the absence of pore formation, was necessary and sufficient for membrane shedding, suggesting that the calcium influx and patch formation were not required for shedding (71). However, the authors did not exclude the possibility that oligomerization and pre-pore formation induced changes in the plasma membrane leading to activation of calcium uptake and other signaling events.

CDC-mediated formation of pores in the plasma membrane leads to many other cell signaling events, which involve apoptosis (72), DNA damage and cell cycle arrest (73), unfolded protein response (74), induction of ubiquitination (75), and transcriptional activation (76, 77).

It should also be mentioned that some CDCs can induce cell activation in the absence of pore formation. The toxins bind to plasma membrane microdomains enriched in cholesterol, gangliosides, GPI-anchored proteins, and Src family kinase called lipid rafts, which are involved in signal transduction (78–80). Using LLO and the cholesterol-inactivated form of LLO (CL-LLO), Gekara et al. demonstrated that both forms of the toxin bind to and induce clustering of lipid rafts (81). Consistent with the role of lipid rafts in cell signaling, the authors found that CL-LLO-induced raft aggregation resulted in activation of tyrosine kinases in a pore-independent manner. The aggregation of rafts could have critical physiological consequences in listeriosis. Under *in vivo* conditions, secreted LLO is inactivated by cholesterol present in body fluids. Although cholesterol-inactivated LLO loses its pore-forming capacity, CL-LLO can activate target cells by aggregating lipid rafts and, in this way, influences the course of *Listeria monocytogenes* infection. In this process, the lectin activity of CDCs, discussed above, could play a key role. Interestingly, LLO was found to bind to carbohydrate structures present on gangliosides (58), which are found in lipid rafts (82).

## CDC Interactions With Mast Cells

Most of the studies focused on the molecular mechanisms of CDC interactions with mast cells were performed using rat, mouse, or human mast cell lines, bone marrow-derived mouse mast cells (BMMCs), or human mast cells isolated from the lungs or intestine. While mechanisms described in previous section are common to various cell types, including mast cells, several mast cell-specific responses are directed towards CDCs. This reflects the unique properties of mast cells, based on transcriptional profiles, dramatically different from other cell types of the immune system (83). This could be in part related to the findings that mast cells are evolutionarily ancient, dating back to at least as far as urochordates (84–86), and that mast cells have unique functions as first-line sentinels of the immune system for host defense against pathogens (87).

### Molecular Mechanism of Mast Cell Activation With CDCs

The canonical way of mast cell signaling through FcϵRI involves, as a first step, ligand (IgE-antigen complexes, lectin, anti-FcϵRI antibody)-mediated aggregation of FcϵRI. The aggregation of the FcϵRI receptors leads to Src family protein tyrosine kinase (PTK) Lyn-mediated phosphorylation of immunoreceptor tyrosine-based activation motives (ITAMs) in the FcϵRI β and γ subunits by incompletely understood mechanism (88). The phosphorylated γ subunits then serve as binding and activation sites for the Syk kinase, which phosphorylates many signaling molecules, including transmembrane adaptor protein LAT1 (linker for activation of T cells) and non T cell activation linker (NTAL or LAT2), reviewed in (89). Phosphorylated LAT1 recruits molecules containing Src homology (SH)2 domains, such as adaptor protein Grb-2 and PLCγ1. An important intermediate is phosphatidylinositol (PI)-3-OH kinase (PI3K), which catalyzes synthesis of PI-3,4-bisphosphate and PI-3,4,5-trisphosphate (PIP3). These phospholipids contribute to the recruitment of PLCγ1 and PLCγ2 and other molecules containing pleckstrin homology domains to the plasma membrane. PLC cleaves the phospholipid phosphatidylinositol 4,5-bisphosphate (PIP2) into diacylglycerol (DAG) and inositol 1,4,5-trisphosphate (IP3). IP3 diffuses through the cytosol to bind to IP3R, Ca^2+^ channels in the ER, and thus causes a rapid but transient release of Ca^2+^ from ER stores to the cytoplasm. Decreased levels of Ca^2+^ in ER are sensed by ER protein STIM1, which then oligomerizes and interacts with the plasma membrane (PM) protein Orai1 at ER–PM junctions [reviewed in (90, 91)]. STIM1-Orai1 assembly forms the active channel responsible for store-operated Ca^2+^ (SOC) channels, which activates the entry of external Ca^2+^ into the cytoplasm. Phospholipids and DAG are used as substrates by phospholipase A (PLA)2 and diacylglycerol lipase, respectively, leading to arachidonic acid production. Arachidonic acid is a precursor in production of eicosanoids such as prostaglandins and leukotrienes, which exert a complex control over many bodily systems, mainly in inflammation and immunity (92, 93). The maintenance and amplification of FcϵRI-generated signals are regulated by the phosphoinositide 3 kinase (PI3K)/Bruton’s tyrosine kinase (Btk) axis (94). This pathway contributes to the cytokine and chemokine production regulation through activation of transcription factors NFAT and NFκB (95). Production of cytokines, chemokines, and other proteins also requires activation of mitogen-activated protein (MAP) kinase pathways and enhanced transcription through the activation of various transcription factors, such as those that bind to promoter regions of the genes encoding the proteins mentioned above. There are three major MAP kinase pathways involving extracellular signal-regulated kinases (ERK), c-Jun N-terminal kinase (JNK), and p38 MAP kinase activated *via* the Ras-Raf pathway in the FcϵRI-activated cells. A key role in this process is played by Ca^2+^ influx, as documented by the possibility to induce degranulation in mast cells by bypassing the early aggregated FcϵRI-mediated events by thapsigargin or calcium ionophore A23187 (96, 97). Significantly, the PI3 kinase is involved in mast cell activation induced through both IgE-dependent and antigen-independent pathways (98, 99).

Based on these and data from other systems showing the involvement of calcium in the activation of transcription factors (67, 100), it has been proposed that CDCs induce mast cell activation by producing pores through which Ca^2+^ enters the cell (70). However, as described by Usmani et al. (101), the interaction of SLO with the cells could increase the concentration of free Ca^2+^ through IP3-mediated depletion of intracellular stores and activation of store-operated Ca^2+^ (SOC) entry dependent on the STIM1-Orai1 cross-talk. This mechanism of Ca^2+^ entry was independent of the toxin’s ability to form Ca^2+^-conducting pores, allowing the cell to respond to much lower toxin concentrations. Thus, SLO may induce IP3 release by a mechanism comparable to the one used by regular surface receptors. The molecular mechanisms of IP3 release by CDCs, however, remain enigmatic. Below, we will review data on three CDCs (SLO, PLY, and LLO) used in the studies on the molecular mechanisms of mast cell activation. In several studies, bacteria producing CDCs and their non-producing forms have been used. Summary data on the effects of CDCs on degranulation, cytokine and chemokine production, tyrosine phosphorylation, and Ca^2+^ responses are presented in **Table 2**. **Table 3** provides summary data on the effect of CDCs on cytokines, chemokines, and other mediators produced in mouse and human mast cells.

**Table 2:** Effect of various cytolysins at sublytic concentrations on mast cell degranulation, production of cytokines, chemokines, and other mediators, tyrosine phosphorylation of signaling molecules, and Ca^2+^ response.

MC response Activating agents	Degranulation			Level of cytokines, chemokines and other mediators		Tyrosine phosphorylation			Ca^2+^ levels
	Histamine release		β-Hexosaminidase release*	mRNA	Protein	P38 MAPK	JNK	Erk1/2	
**Cytolysins**									
SLO		↑ (102)		↑ (102)	↑ (102)	↑ (102)	↑ (102)		
		↑ (103, serotonin*)							
PLY	− (104)				↑ (104)				
LLO		↑ (70)		↑ (70)	↑ (70)				↑ (70)
Cholesterol-inactivated LLO		− (70)		− (70)					− (70)
**Cytolysin-producing and non-producing bacteria**									
SLS producing *S. equi* live bacteria	↑ (105)			↑/− (106)	↑ (105, 106)	↑ (106)			
SLS non-producing *S. equi* live bacteria				− (106)	− (106)	− (106)		− (106)	
PLY producing *S. Pneumoniae* live bacteria (107) lysed bacteria (102, 108)	− (104)	↑ (26, 109)			− (109)				
					↑/− (26) ↑ (104)				
PLY-non-producing *S. pneumoniae*	− (104)	− (26)			− (26, 104)				
LLO-producing *Listeria monocytogenes*		↑ (70)			↑ (70, 110)				↑ (70)
		↑ (110, CD107a*)							
LLO-non-producing *Listeria monocytogenes*		− (70)							− (70)
Others									
Saponin				↑ (106)	↑ (106)	↑ (106)		↑ (106)	

↑Increased level, − unchanged level.

*In these studies, serotonin release or CD107a surface expression were used for quantification of degranulation instead of β-hexosaminidase release.

**Table 3:** Effect of cytolysins or bacteria producing the corresponding cytolysins on production of selected cytokines, chemokines, and other mediators by various mast cell types.

Mast cell type	Changes in production of cytokines, chemokines, and other mediators in response to cytolysins or bacteria producing the corresponding cytolysins		
	At the mRNA level	At the protein level	Ref.
**Mouse**			
BMMC	↑: TNF-α, IL-4, IL-6, IL-13, GM-CSF, MCP-1, Nr4a3	↑: TNF-α, IL-2, IL-4, IL-5, IL-6, IL-12, IL-13, CCL2/MCP-1, CCL3, CCL4, CCL5, RANTES, GM-CSF	(26, 70, 102, 105, 106, 110)
		−: TNF-α, IL-1β, IL-4, IL-10, IL-12, IL-17A, IFN-γ, TGF-β	
BMMC (response to SLS-deficient strain)	−: TNF-α, IL-6, Nr4a3	−: TNF-α, IL-6, CCL2/MCP-1	(106)
BMMC (response to PLY-deficient strain)		−: IL-6, -CCL2/MCP-1	(26)
PCMC	↑: IL-6, Nr4a3	↑: TNF-α, IL-6, CCL2/MCP-1	(106)
	−: TNF-α		
PCMC (response to SLS-deficient strain)	−: TNF-α, IL-6, Nr4a3	−: TNF-α, IL-6, CCL2/MCP-1	(106)
**Human**			
HLMC		↑: LTC4	(104)
Human intestinal MC	↑: TNF-α, IL-3, -5, -6, CXCL8	↑: TNF-α, CXCL8, LTB4, sLT	(111)
Human intestinal MC (response to Hly-deficient strain)	−: TNF-α, IL-5, CXCL8	−: LTB4, sLT	(111)
HMC-1		↑: LTC4	(104)
HMC-1 (response to PLY-deficient strain)		−: LTC4	(104)
LAD2		↑: LTC4	(104)

↑ increased level.

− unchanged level.

BMMC, Bone marrow-derived mast cells.

PCMC, Peritoneal cell-derived mast cells.

HLMC, Human lung mast cells.

HMC-1, Human mast cell line.

LAD2, Human mast cell line.

### Streptolysin O (SLO)

SLO is a multifunctional protein with pore-dependent and pore-independent functions (112, 113) produced by *Streptococcus pyogenes*. This pathogen is responsible for various infectious diseases, including pharyngitides, rheumatic fever, scarlet fever, and life-threatening conditions such as necrotizing soft tissue infection (necrotizing fasciitis) and streptococcal toxic shock syndrome (23, 24). In initial studies, peritoneal mast cells were permeabilized by treatment with SLO at concentrations that generated membrane lesions. The permeabilized mast cells released histamine and β-N-acetylglucosaminidase, dependent on the presence of nucleoside triphosphate and micromolar concentrations of Ca^2+^. SLO-permeabilized mast cells have been used as a simplified system for studies to understand the molecular mechanisms of exocytosis (114–118) and the role of plasma membrane repair mechanisms (65, 76, 119). In these experiments, relatively high SLO doses were used, allowing formation of numerous pores in the plasma membrane of target cells. While such doses are cytocidal, low doses are tolerated because a limited number of lesions are formed and can be resealed by repair mechanisms (120).

Further studies showed that sublethal doses of SLO rapidly activated BMMCs to degranulate and to induce production of mRNAs of several cytokines, including tumor necrosis factor (TNF)-α, IL-13, IL-4, IL-6, and GM-CSF (**Figures 3** and **4**). Production of TNF-α was blunted upon pharmacological depletion of protein kinase C by phorbol-12 myristate-13 acetate. Exposure to low, nontoxic concentrations of SLO also resulted in enhanced phosphorylation of stress-activated protein kinases p38 mitogen-activated protein (MAP) kinase and c-jun N-terminal kinase (JNK). Inhibition of p38 MAP kinase markedly reduced production of TNF-α, suggesting that transcriptional activation of mast cells following transient permeabilization might contribute to the host defense against streptococcal infections *via* the beneficial effects of TNF-α. However, mast cell hyperstimulation might also lead to overproduction of TNF-α, promoting development of the toxic streptococcal syndrome (102). The involvement of TNF-α in the host defense against streptococcal infection was described in previous studies (13, 76, 107, 108).

The resistance to CDCs is affected by the levels of surface cholesterol and proteins exposed to the plasma membrane. Schoenaurer et al. (121) described that toxin-induced plasma membrane perforation caused by SLO in human mast cells (HMC1) is affected by expression levels of the P2X7 receptor. The P2X7 receptor is an ATP-gated trimeric membrane cation channel, which after activation with ATP triggers Ca^2+^ influx and induces blebbing (122). This protective effect of the P2X7 receptor can be increased by activating the P2X7 receptor with ATP and abolished by selective P2X7R antagonists, A-438079 or blebbistatin (121).

Other studies showed that SLO is not the only activator of the mast and other immune cells, but also inhibits immune cell activity. Experiments with neutrophils revealed that SLO sublytic concentrations suppressed the oxidative burst in neutrophils, facilitating bacteria escape from innate immune killing (123). Thus, SLO functions as a virulence factor that is necessary and sufficient for suppressing bactericidal ROS production, thereby subverting neutrophil ROS-dependent killing. Interestingly, S. aureus, producing pore-forming virulence factor α-hemolysin (H1a), did not modulate the oxidative burst of neutrophils, suggesting a specific unique role of SLO, not a general consequence of membrane perturbation and disruption. Further studies showed that LLO and PFO could suppress the oxidative burst in murine macrophages by preventing NADPH oxidase localization with phagosome (124). In addition to oxidative burst suppression, low concentrations of SLO prevented release of IL-8 and elastase and blocked formation of neutrophil extracellular traps (123). It should also be mentioned that Logsdon and colleagues (113) showed that SLO at low levels reduced bacteria internalization by keratinocytes and concluded that SLO interferes with the internalization through local perturbations of the cell membrane and disruption of clathrin-dependent uptake pathways. They suggested that by forming plasma membrane pores in cholesterol-rich membrane domains, SLO may mimic cholesterol depletion and, in this way, inhibit the clustering of lipid rafts, thereby interfering with integrin signaling and bacterial internalization. These results are compatible with a model in which SLO binding (through fibronectin) to integrins of the cell surface acts to cluster integrin-containing membrane domains, thereby enhancing integrin-mediated cell signaling to stimulate the process of bacterial internalization. The results obtained *in vitro* were confirmed by experiments *in vivo* in which injection of SLO into ears of mast cell-deficient mice (KitW/KitW-v) resulted in a weak inflammatory response when compared to KitW/KitW-v mice that had been selectively engrafted with BMMCs (103).

### Pneumolysin (PLY)

Most pathogenic isolates of *Streptococcus pneumoniae* produce PLY. These bacteria are the leading source of bacterial pneumonia and could cause otitis media and bacterial meningitis (25, 26). Experiments *in vitro* showed that exposure of rat RBL-2H3 cells to various strains of *S. pneumoniae* leads to degranulation in a dose- and time-dependent manner. The degranulation was only partially controlled by cytosolic calcium and was not accompanied by production of TNF-α and IL-6 (109). The authors suggested that the induction of mast cell degranulation by pneumococcal factors not accompanied by the production of proinflammatory cytokines may be a specific strategy elaborated by this bacterium to promote its spreading from the respiratory mucosa and reducing neutrophil infiltration. Thus, the PLY amount could be meager, and other bacterial proteins could play a dominant role.

Human lung mast cells (HLMCs) and human mast cell lines HMC-1 and LAD2 were cocultured with pneumococci or stimulated with PLY. HLMCs and cell lines exhibited antimicrobial activity against *S. pneumoniae*. PLY induced release of antimicrobial peptide cathelicidin LL-37. These data suggested that mast cells limit pneumococcal dissemination early in the course of pneumococcal pulmonary disease (104). A recent study extended these findings and showed that mouse BMMCs degranulated and released IL-6, CCL2, CCL3, and CCL4 (but not IL-1β, TNF-α, IFN-γ, and several other cytokines) after exposure to *S. pneumoniae* (104, 125).

Furthermore, the response of BMMCs varied among different pneumococcal serotypes and was dependent on PLY but independent of TLR activation (26). These studies suggested that the absence of mast cells or pharmacologic mast cell stabilizer (cromoglycate) may reduce inflammation and ameliorate the disease severity following intracisternal infection in mice with *S. pneumoniae*. Surprisingly, experiments *in vivo* using mast cell-deficient strains (WBB6F1-KitW/Wv and C57BL/6 KitW-sh/W-sh mice) showed no significant effect on the disease phenotype of experimental pneumococcal meningitis (26). Thus, the results do not support previous *in vivo* data showing that mast cell-deficient KitW-sh/W-sh mice exposed to *S. pneumoniae* exhibited reduced inflammation, lower bacterial outgrowth, and longer survival compared with wild-type (WT) mice (126).

### Listeriolysin (LLO)

Pore-forming toxin LLO is the main virulence factor of *Listeria monocytogenes*. LLO is known to induce a broad spectrum of host responses that ultimately influence the outcome of listeriosis. Unlike the other pathogens producing CDCs, *Listeria monocytogenes* is an intracellular pathogen that requires its CDC, LLO, for intracellular survival. LLO is thus the only cytolysin that is secreted by a facultative intracellular pathogen, while all other CDCs are produced by pathogens that are largely extracellular. LLO monomers bind to plasma membrane microdomains in target cell membranes, dimerize and oligomerize to form pre-pore complexes, followed by formation of pores of 50-80 subunits. Formation of pre-pores leads to aggregation of lipid rafts and signal transduction. Formation of pores and reparation processes are other steps of CDC-mediated cell activation in which extracellular components (Ca^2+^) enter the cytoplasm, and cytoplasmic components (such as K^+^ and proteins) are released from the cell. This leads to signals in target host cells resulting in degranulation, cytokine and chemokine production, suppression of phagocytosis, and induction of apoptosis (127–130) (**Figures 3** and **4**).

Using BMMCs and RBL-2H3 cells, Gekara et al. (70) showed that LLO triggers cellular responses such as degranulation and cytokine synthesis in a Ca^2+^-dependent manner. They also found that LLO-mediated Ca^2+^ signaling is due to Ca^2+^ influx from the extracellular milieu and release of Ca^2+^ from intracellular stores. Ca^2+^ release from intracellular stores occurs *via* activation of intracellular Ca^2+^ channels, which involve tyrosine phosphorylation of several proteins, including PLC-γ1 and IP_3_R-operated Ca^2+^ channels activated *via* G-proteins and protein tyrosine kinases. These data and the fact that the Ca^2+^ release could partially be blocked by tyrosine kinase inhibitor genistein and G-protein inhibitor pertussis toxin suggested that LLO activated the IP_3_R Ca^2+^ channels *via* tyrosine phosphorylation and G-protein activation of PLC γ and PLC β isoforms, respectively. Another mechanism of Ca^2+^ release from intracellular stores is Ca^2+^ channel independent, which could reflect injury of intracellular stores, such as the ER (70). The data are relevant to previous studies showing that exposure of human embryonic kidney cells to sublytic concentrations of LLO caused long-lasting oscillation of the intracellular Ca^2+^ levels, leading to a pulsed influx of extracellular Ca^2+^ through pores that LLO forms in the plasma membrane. Calcium influx did not require the activity of endogenous Ca2+ channels. These data indicated that Ca^2+^ oscillations modulate cellular signaling and gene expression and could form a basis for the broad spectrum of Ca^2+^-dependent cellular responses induced during *Listeria* infection (68).

Later studies using macrophage cell line J774 indicated that LLO is a potent aggregator of plasma membrane components, including GPI-anchored proteins CD14, CD16, ganglioside GM1, and protein tyrosine kinase Lyn. Abrogation of the cytolytic activity of LLO by cholesterol pretreatment was found not to interfere with the ability of LLO to aggregate the above-mentioned surface molecules nor to trigger tyrosine phosphorylation of Lyn and Syk kinases in the cells. When the oligomerization of LLO was blocked by monoclonal anti-LLO antibody, aggregation of surface molecules and tyrosine phosphorylation were blocked. The combined data suggested that LLO induces signaling through coaggregation of the plasma membrane receptors, kinases, and adaptors (81). Recently discovered lectin activity of LLO could play a vital role in this process (58). However, cholesterol-inactivated LLO, which binds and aggregates plasma membrane components such as the active form of LLO, could not induce Ca^2+^ release in mast cells. Thus, it is likely that membrane binding or plasma membrane protein aggregation is not sufficient to activate the IP_3_R-dependent pathways and that LLO oligomerization and transmembrane insertion leading to pore formation are essential in this process (70).

Contrary to the study of Gekara et al. (70), Jobbings et al. (110) found that in the absence of Ca^2+^, LLO-mediated degranulation was enhanced, whereas antigen and PMA/I-mediated degranulation was completely inhibited. This discrepancy could be explained by LLO-mediated pore formation in granules resulting in degranulation and mediator release. Thus LLO is required for mast cell degranulation, independent of extracellular Ca^2+^. The authors also found that in mast cells, LLO induces transient downregulation of cell surface c-kit receptor (CD117) without any effect of the FcϵRI expression. Detailed analysis showed that in response to *L. monocytogenes*, mast cells release in addition to the key inflammatory cytokines (TNF-α and IL-6) a range of other mediators including Osteopontin, IL-2, IL-4, IL-13, granulocyte-macrophage colony-stimulating factor (GM-CSF), and several chemokines (CCL2, CCL3, CCL4, and CCL5). These cytokines are released in a MyD88-dependent manner.

A recent study (51) showed that four D4 subunits of LLO in the membrane-bound state are placed in the bilayer interface in a pre-pore configuration. In contrast, the membrane-inserted state consists of a tetrameric arc-like pore configuration. The binding of LLO leads to induced spatial heterogeneity that occurs in both membrane-bound and membrane-inserted states. This heterogeneity is primarily driven by the local density enhancement of cholesterol in the vicinity of LLO D4 subunits in the membrane-bound state. The induced heterogeneity after plasma membrane binding of LLO could be at least in part responsible for the observed changes in signaling machinery in cells exposed to low concentrations of CDCs. In this process, aggregation of lipid raft components (**Figure 2**) could play an important role.

## Comparable Response of Mast Cells to CDCs and Other Pore-Forming Compounds

The results so far discussed indicate that various CDCs at sublytic concentrations induce similar mast cell activation events. This could reflect the structural similarity of CDCs examined (**Figure 1**) or the general effect of compounds leading to plasma membrane perturbation. Several lines of evidence indicate that the mast cell inflammatory response described for CDCs is comparable to other pore-forming and membrane destabilizing compounds.

Extensive studies have been devoted to SLS produced by *Streptococcus equi*, which causes a highly contagious and common disease of the upper respiratory tract and associated lymph nodes in equids (29). SLS was also identified in *Streptococcus pyogenes* and other *Streptococcus* species. Genomic analysis has identified gene clusters that are similar to the SLS-associated cluster in other pathogens such as *Listeria monocytogenes*, *Clostridium botulinum* and *Staphylococcus aureus* (131). SLS belongs to a distinct group of toxins whose hemolytic activity is sensitive to trypan blue, which are resistant to cholesterol and unaffected by oxidation. In these properties it differs from SLO and other CDCs (29, 131, 132). An initial ultrastructural study using BMMCs as a model showed the extensive formation of dilated ER in response to *S. equi* exposure, indicating enhanced protein synthesis (105). Further analysis revealed that exposure of BMMCs to *S. equi* did not show signs of extensive degranulation. However, the coculture of live bacteria with BMMCs resulted in profound secretion of IL-4, IL-6, IL-12, IL-13, TNF-α, CCL2, CCL7, MCP3, CXCL2, CCL5. In contrast, heat-inactivated bacteria caused only minimal cytokine/chemokine response (105).

A recent study showed that BMMCs responded vividly to wild-type *S. equi* by upregulating a panel of proinflammatory genes and secreting proinflammatory IL-6, TNF-α, and monocyte chemoattractant protein (MCP)1 (106). However, this response was abrogated entirely in *S. equi* lacking the sagA gene encoding SLS. Several lines of evidence indicated that mast cell activation is not the result of mast cell lysis and release of components capable of mast cell triggering. Immunoblotting analysis revealed that exposure of mast cells to wild-type *S. equi*, but not to a SLS-deficient mutant, induced phosphorylation of p38 and Erk1/2, which could be inhibited by the corresponding inhibitors. Based on these data, the authors concluded that bacteria-derived SLS at sublytic concentrations is a major stimulus for mast cell activation leading to proinflammatory gene expression and cytokine production. It should be noted, however, that in contrast to CDCs, SLS induced only week degranulation (106).

Kramer et al. (111) examined the effect of α-hemolysin, a protein toxin that assembles on membranes to form a heptameric pore structure (133). They found that *Escherichia coli* strains producing α-hemolysin induced release of histamine, leukotrienes, and proinflammatory cytokines from intestinal human mast cells. Blocking the extracellular Ca^2+^ and calmodulin/calcineurin pathway by cyclosporine A inhibited the response to α-hemolysin. Activation of mast cells by α-hemolysin was also inhibited by blocking MAPKs p38 and ERK. Pharmacological blockade of Ca^2+^-dependent PKCα, and PKCβ1 or PI3K had an only weak effect on α-hemolysin activation of mast cells, but a robust inhibitory effect on FcϵRI-mediated cell activation. The data indicate that mast cell activation by FcϵRI and α-hemolysin utilize different signal transduction pathways (111).

During their experiments focused on *S. equi* interaction with mast cells, von Beek et al. (106) used another pore-forming compound, saponin, and a steroid glycoside detergent digitonin to determine whether SLS and saponins trigger BMMCs in a similar way. They found that saponin, which forms tiny pores in the plasma membrane (134, 135) at sublytic concentrations, triggered IL-6 and TNF production similarly to SLS. They concluded that mast cell activation by *S. equi* SLS could be phenocopied by low sublytic concentrations of saponin. When steroidal saponin, digitonin, was used at sublytic concentrations, profound production of IL-6 in mast cells was also observed. Altogether, these data suggest that multiple lytic agents at sublytic concentrations could induce mast cell activation by similar mechanisms.

## Conclusions and Future Directions

Despite impressive progress in understanding the molecular mechanism of CDCs’ interaction with plasma membranes and CDC-mediated activation of the mast and other cell types, the picture is far from complete. The involvement of similar signaling pathways triggered by CDCs and saponins suggests a similar cell response towards plasma membrane perturbations. These perturbations involve (I) binding of ligands to plasma membrane structures (GPI-anchored proteins, cholesterol-rich domains, glycoconjugates), (II) aggregation of monomers (oligomerization process) and pre-pore formation, (III) plasma membrane pore formation, and (IV) activation of the signaling machinery leading to cell activation and production of inflammatory mediators. The molecular-level details of individual steps are yet to be determined. Although studies of membranes can now benefit from the large-scale detailed analysis of lipid molecular species, there is currently a paucity of data regarding some of the critical points, such as (I) lipid compositional analysis of plasma membrane domains from cells at various stages of pore formation and (II) complete compositional analysis of proteins associated with lipid pre-pores and pores at various time intervals after exposure to CDCs. Furthermore, a comparison of phosphoproteomes from cells activated *via* IgE-antigen complexes, sublytic concentrations of CDCs, or sublytic concentrations of saponins could be informative in delineating specific signaling pathways involved in cell activation by CDCs.

Most of the experiments described in this review were performed *in vitro*. Models that more closely resemble *in vivo* conditions are needed to unravel the relevance of CDCs and other pore-forming compounds to the pathogenesis of various diseases caused by CDC-producing bacteria. Such studies will be of great value in rational usage of CDCs as anti-cancer therapeutics (136, 137), vaccine adjuvants (138–141), and adjuvants stimulating inflammasome activity (142).

## Author Contributions

LD, MT, and PD wrote the manuscript. All authors contributed to the article and approved the submitted version.

## Funding

This work was supported by projects 18-18521S and 20-16481S from the Czech Science Foundation and by institutional project RVO 68378050.

## Conflict of Interest

The authors declare that the research was conducted in the absence of any commercial or financial relationships that could be construed as a potential conflict of interest.
